# Highly variable lifespan in an annual reptile, Labord’s chameleon (*Furcifer labordi*)

**DOI:** 10.1038/s41598-017-11701-3

**Published:** 2017-09-12

**Authors:** Falk Eckhardt, Peter M. Kappeler, Cornelia Kraus

**Affiliations:** 1 Dept. Sociobiology/Anthropology, Institute of Zoology and Anthropology, University of Göttingen, Kellnerweg 6, 37077 Göttingen, Germany; 2 Behavioral Ecology and Sociobiology Unit, German Primate Center, Kellnerweg 4, 37077 Göttingen, Germany; 3 Wissenschaftskolleg zu Berlin, Wallotstr. 19, 14193 Berlin, Germany

## Abstract

Among tetrapods, the current record holder for shortest lifespan is Labord’s chameleon, *Furcifer labordi*. These reptiles from the arid southwest of Madagascar have a reported lifespan of 4–5 months during the annual rainy season and spend the majority of their life (8–9 months) as a developing embryo. This semelparous, annual life history is unique among tetrapods, but only one population (Ranobe) in the southernmost distribution range has been studied. We therefore investigated the potential for environmentally-dependent variability in lifespan in a population in Kirindy Forest, which has a much longer warm rainy season. While no adults were found after March in Ranobe, the disappearance of adults was delayed by several months in Kirindy. Our data also revealed sex-biased mortality, suggesting that females have a longevity advantage. Furthermore, we found that, after an unusually long previous rainy season, one female was capable of surviving until a second breeding season. Keeping *F. labordi* in cages under ambient conditions demonstrated that also males can also survive until the next season of activity under these conditions. Our study therefore revealed considerable variability in the extreme life history of this tetrapod that is linked to variation in ecological factors.

## Introduction

The fast-slow continuum is the dominant axis of life-history variation in tetrapods. Fast-living species are characterized by fast growth, high reproductive rates, high aging rates and short lifespans, compared to their slow-living counterparts^1–3^. Among tetrapods, the most extreme example for short lifespan is provided by Labord’s chameleon, *Furcifer labordi*. During their study in the arid southwest of Madagascar in Ranobe, Karsten *et al*.^4^ reported synchronous hatching of *F. labordi* with the onset of the annual rainy season in November. Here, early life of this chameleon is characterized by fast growth, resulting in sexual maturity at less than two months of age. After mating, senescent decline becomes apparent, and by the end of the rainy season in March, a population wide die-off of both sexes occurs. Thus, with an incubation period of 8–9 month, *F. labordi* spend the majority of their lifetime as a developing embryo in the egg, probably as an adaption to the highly seasonal climate. High adult mortality combined with relatively high juvenile survival might have ultimately selected for this semelparous, annual life history^5, 6^.

Semelparity, the strategy to invest in only one mating event, is rare among tetrapods, including a few small-sized marsupial species from the families Didelphidae and Dasyuridae. However, in these marsupials the die-off following the mating season is restricted to males, while some females survive to breed a second time^7^. Strong prey seasonality leading to a short breeding season has been proposed to explain obligate male semelparity in these marsupials^8^. Interestingly, males that were captured before the mating season and prevented from competing for mates survived for more than two years^9, 10^. Less extreme cases of semelparity among marsupials have also been described. Here, facultative male die-off in the wild is restricted to some populations and/or only observed in some years and linked to variable resource availability due to variable climatic conditions^11, 12^.

*Furcifer labordi* has so far only been studied in the southernmost and driest part of its distribution range^4^. We therefore conducted a field study of a population in Kirindy Forest, which is situated near the northern distribution range and characterized by a longer annual rainy season, to investigate potential intraspecific variation in lifespan. Since Madagascar is known for its high unpredictability in inter-annual rainfall^13^, we also focused on differences in lifespan due to environmental variation between years. Additionally, we examined differences in sex-specific mortality because mortality varies by sex in some semelparous marsupials^8^. To characterize intrinsic rates of aging, we excluded extrinsic factors of mortality due to predation, costs of reproduction, fighting as well as water and food shortage by keeping some individuals of both sexes in field cages.

## Results

### Capture-mark-recapture study

**Figure 1:**
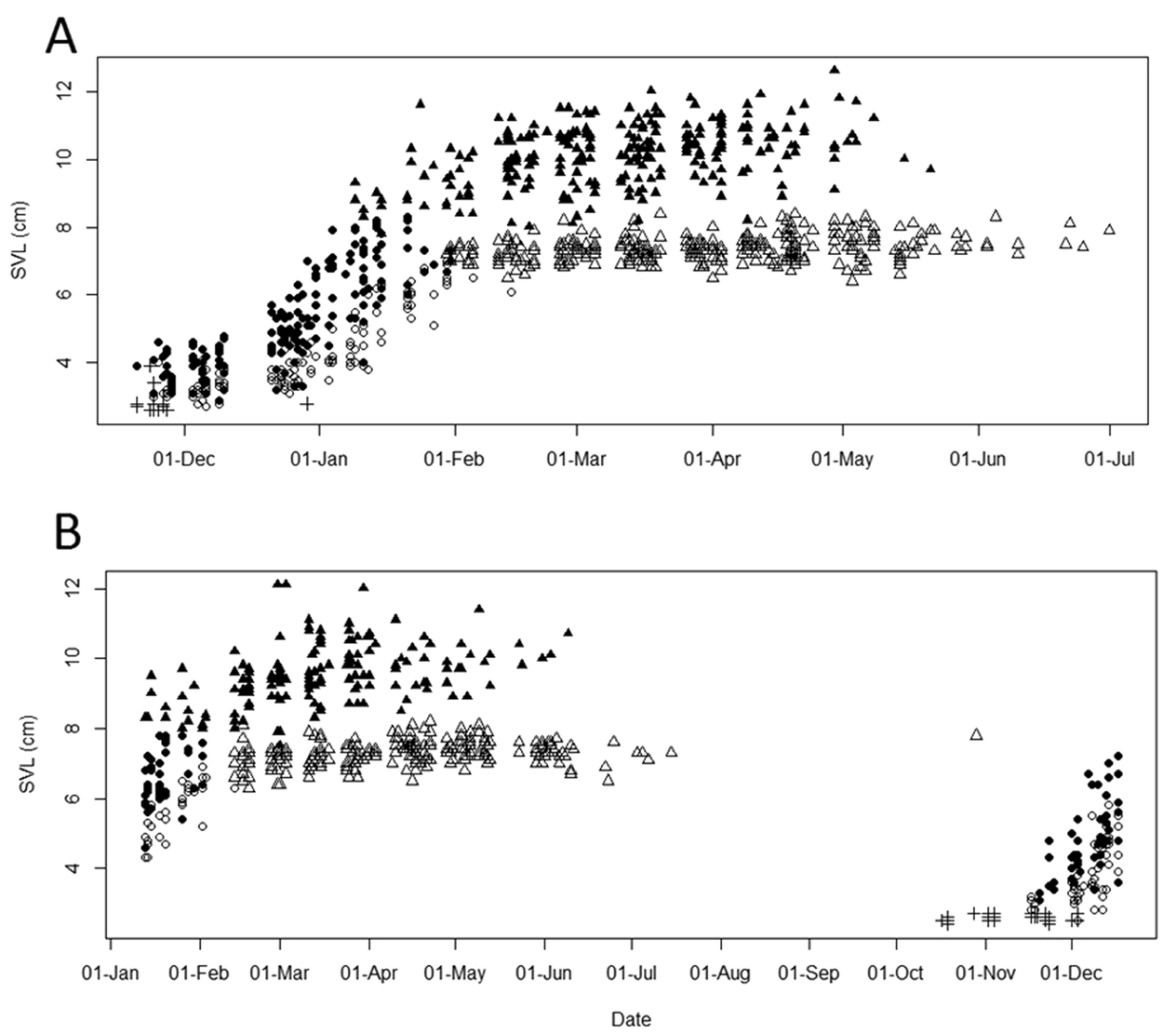
Growth curves of *F. labordi* in Kirindy Forest. Depicted is SVL against date: unsexed hatchlings (+); males (filled symbols); females (open symbols), juveniles (circles), and adults (triangles). (**A**) Data of field season from November 19, 2013–July 9, 2014 (n = 881), (**B**) Data of field seasons from January 11 – July 2015 (n = 439), and October 12 – December 17, 2015 (n = 142).

Hatchlings were mainly found between mid-October and early December. Recaptures of marked individuals allowed us to estimate average daily juvenile growth rates for males (1.37%, *n* = 13, 0.76 ± 0.48 mm; mean ± SD) and females (1.18%, *n* = 12, 0.55 ± 0.27 mm; mean ± SD). We first found adult males in early January and adult females in late January. Average snout-ventral length (SVL) for adult males was 100.3 ± 8.62 mm (n = 344) and for females 73.3 ± 3.7 mm (n = 500). Gravid females appeared from the end of January onwards. Later in the reproductive season, we found that some females could reproduce more than once (n = 3, 4.6% of all re-captured adult females). We assumed a gestation period of maximally four weeks and recaptured these females at least five weeks after we noticed that they were gravid. Additionally, later in the reproductive season several gravid females (n = 48) showed abrasion on their wrist. These marks most likely resulted from excavation for the deposition of a previous clutch. While the sex ratio in juveniles was almost even, the adult sex ratio became highly female-biased across the reproductive season in 2014 (Feb to May-Jul: *χ* ^2^ = 39.2, *df* = 3, *P* < 0.001) and 2015 (Feb to Jun-Jul: *χ* ^2^ = 48.2, *df* = 4, *P* < 0.001). In the season 2013/14, no males were found after May 27, while single females were detected until July 1 (Fig. 1A). In contrast, in the reproductive season 2015, males were detected until June 9, and several females were active until the end of the field season in mid-July, when some were still in good physical condition and even gravid (Fig. 1B). In total, recapture rates were rather low (98 out of 881 captures; 11.12%) in the season 2013/14 and (33 out of 439 captures, 7.52%) in the second season 2015 and (2 of 142 captures, 1.41%) in the third field season. At the beginning of the third field season, we encountered an adult female originating from the previous active season on October 29. This animal was in good physical condition (Fig. 1B). For further observation, we kept her in captivity. In December, she was put together with an adult male, which had been kept alive in captive field conditions, resulting in immediate mating. This female survived until March 2016, a presumed lifespan of 16 months.

### Experimental field enclosures – Keeping *F. labordi* in protected field conditions

**Figure 2:**
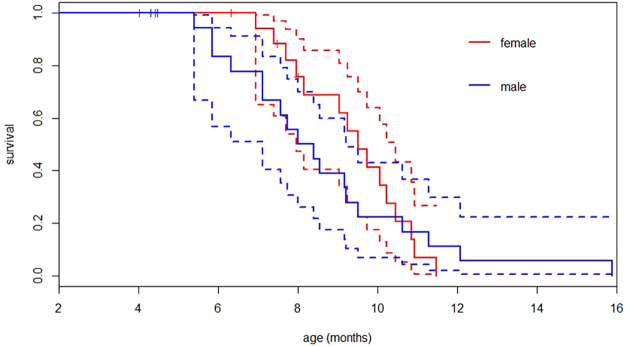
Kaplan-Meier survival curves of *F. labordi* in captivity. Continuous lines indicate probability of survival of males (blue) and females (red). Dashed lines represent corresponding 95% confidence interval. Small vertical bars show censored events due to escape or death by egg-binding.

We found no significant differences in survival probability between captive males and females (logrank-test: *χ* ^2^ = 0.3, *df* = 1, *P* = 0.59). Median survival time for females was 9.5 months and for males 8.2 months. Maximum lifespan for females was 11.5 months and for males 16 months. Two males and two females escaped from damaged cages during a cyclone. Moreover, three females died because of egg binding (Fig. 2).

## Discussion

Our study revealed considerable variability in the life history of *F. labordi*, suggesting that their survival in nature is to some degree responsive to ecological conditions. During an average rainy season in 2013/14, lifespan ranged from 6–9 months and was therefore longer compared to longevity reported for the southern population. Both populations also differed in several aspects of their life history, with all stages of life being more extended at Kirindy. Karsten *et al*.^4^ reported synchronous hatching and rapid daily juvenile growth rates of 1.86% in SVL in both sexes reaching maturity at less than 60 days. Hatchlings emerged around four weeks earlier at Kirindy and we observed slower juvenile growth rates, resulting in first sightings of adult males after 79 and adult females after 105 days, counted from the detection of first hatchlings. Karsten *et al*.^14^ hypothesized that females produce only one clutch in their lifetime. We recaptured females that were gravid at least twice, suggesting that females in Kirindy may lay more than one clutch and, hence, are not strictly semelparous. Furthermore, both populations show a considerable difference in male adult body size (SVL, Kirindy: 100.3 ± 8.62 mm, n = 344; Ranobe: 87.3 ± 1.3 mm, n = 99^15^. All these differences might be linked to higher annual precipitation levels and longer vegetation periods at Kirindy (900 mm) compared to Ranobe (420 mm)^4^.

We reviewed climate data for Kirindy (2005–2015) and found that rainfall during the second field season was, compared to the long-term average, exceptionally high, resulting in an extended vegetation period. Consequently, members of both sexes were detected about 2 weeks longer (minimum estimate for females), and one marked female was even found during the following active season. Thus, in rare cases, a very small proportion of adult females may survive the dry season under favourable environmental conditions and they may even enter the next reproductive season.

Our data revealed a remarkable difference in sex-specific mortality, indicating that females have a longevity advantage somewhat similar to semelparous marsupials^7^. The obligate male die-off after reproduction in nature is likely related to intense intra-sexual competition^14, 15^, as many adult males showed wounds or scars, especially at the head. Karsten *et al*.^15^ reported potentially receptive females being very selective in terms of mate choice, compared to a larger perennial species. Intense intra-sexual selection might have led to physically intense male encounters with increasingly females-biased adult sex ratios. Additionally, in several other chameleon species, mate guarding was observed, and some males did not feed for several days^16^. We detected numerous males roosting close to females, which is suggestive of mate guarding in this species as well.

Keeping *F. labordi* in captive field conditions revealed that both sexes could outlive their conspecifics in the wild, showing that senescence can be delayed. Moreover, no significant differences in sex-specific mortality were found under these sheltered conditions. However, two males showed extended survival (12 and 16 months) compared to free-living conspecifics. These observations resemble that of some male semelparous marsupials, where lifespan was dramatically expanded by capturing them before the mating season^9, 10^. Moreover, one of the *F. labordi* males was able to mate at the age of 13 months. Hence, lifespan might strongly depend on external causes of mortality that were excluded in captivity.

Despite the remarkable plasticity in lifespan of *F. labordi*, the accelerated life history remains unique and several aspects favouring such a short lifespan remain unknown. Because *F. labordi* has perennial sympatric congeners, this chameleon assemblage constitutes a promising model for comparative investigations of the ecological and physiological determinants of longevity and senescence in wild tetrapods^4^.

## Materials and Methods

### Study site

Kirindy Forest is a dry deciduous forest, in Western Madagascar (44°39′E, 20°03′S, 30–60 m above sea level). Climate is characterized by a hot rainy season (November – March, with on average 900 mm annual precipitation), followed by a cool dry season from (April-October)^17^. Sampling took place over three field seasons: November 18, 2013 – July 9, 2014, January 11 – July 15, 2015, and October 12, 2015 – December 17, 2015.

### Ethics statement

All applicable international, national, and institutional guidelines for the capture and keeping of animals were followed. Research protocols, capture procedures, and keeping of chameleons were approved and permitted by the Ministry for the Environment, Water and Forests of Madagascar, MINEEF.

#### Capture-mark-recapture

*Furcifer labordi* were located at night using flashlights. They often roost on distal branches at a height of 0.5–3 m, and were therefore relatively easy to detect. Chameleons were either captured by hand or removed from higher branches using a stick to which they were encouraged to grip. We sampled alternating along two transects of 3 km length each. We had a recurring order of 10 sampling nights with a break of four nights within each field season. Each capture location was marked by a flag and a GPS recording was taken. Animals were taken to the research camp and handled the following morning. They were sexed and their age categorized (hatchling, juveniles and adults), and their snout-vent-length (SVL) was measured. Hatchlings were identified by the little opening of the abdominal wall due to the former connection with the yolk sack, which is visible during the first days after hatching. Growing individuals were assigned juvenile status until they were sexually mature. Female chameleons were identified as adult when the colorful mating patterns appear and males by the presence of a distinct hemipenis bulge and a hard, ossified rostral appendage. Chameleons were individually marked by visual implant elastomers (VIE; Northwest Marine Technology Inc., Shaw Island, WA). Hatchlings and small juveniles were individually marked by nail polish on the toes. Animals were released at their point of capture the next day. We measured juvenile growth rates and estimated adult sex ratios. To determine growth rate, we calculated daily growth rate from juveniles between their first capture and subsequent re-capture. We compared sex ratios monthly throughout the reproductive season using a *χ* ^2^-test.

#### Experimental housing

We collected juveniles in early January at around two months of age in the forest outside the transect system to exclude any influence on the study population. In total, 20 males and 20 females were housed separately without visual contact in cylindrical outdoor enclosures made of nylon screen (90 cm height, 60 cm diameter). The enclosures were equipped with branches and artificial plants. They were placed in a large outdoor cage in the forest, close to the research camp. Thus, they experienced largely the same temperature and daylight conditions as their conspecifics in the wild. Chameleons received a standardized amount of insect food, depending on age and size to match growth and final size of free-ranging animals. Specifically, chameleons were fed five times per week with two crickets, grasshoppers or butterflies. Water was offered daily with a spray flask. We used the Kaplan – Meier estimator^18^ to assess survival probability of both sexes in captivity.

## Electronic supplementary material

Dataset 1
